# An education intervention to improve health literacy and decision making about supporting self-care among older Australians: a study protocol for a randomised controlled trial

**DOI:** 10.1186/s13063-017-2182-2

**Published:** 2017-09-26

**Authors:** Caroline A. Smith, Esther Chang, Gisselle Gallego, Lynda G. Balneaves

**Affiliations:** 1 National Institute of Complementary Medicine, Western Sydney University, Locked Bag 1797, Penrith, NSW 2571 Australia; 2 School of Nursing and Midwifery, Western Sydney University, Locked Bag 1797, Penrith, NSW 2571 Australia; 3 School of Medicine, The University of Notre Dame, Sydney, Australia; 4 College of Nursing, Rady Faculty of Health Sciences, University of Manitoba, 89 Curry Place, Winnipeg, Manitoba R3T 2N2 Canada

**Keywords:** Complementary medicine, Randomised controlled trial, Health literacy, Australia

## Abstract

**Background:**

Older Australians are high consumers of complementary and alternative medicines (CM). To help older people to take an active role in their health, we will develop and evaluate a novel educational intervention to support decision self-efficacy, and improve health literacy skills.

**Methods:**

The primary hypothesis is that participants receiving a web/DVD plus booklet intervention compared with a booklet-only group will demonstrate an increase in decision self-efficacy. This study is a randomised controlled trial. One hundred and sixty-eight people aged 65 years and older will be recruited from community settings comprising retirement villages and community groups, based in Sydney, Australia. Participants will be randomly allocated to either the education intervention delivered by the Internet or a DVD plus booklet versus a control group (booklet only). The primary outcome measure is CM decision self-efficacy. Secondary outcomes are health literacy, knowledge and attitudes, and change in health-seeking behaviour. Participants’ views on the ease of using the resources, the length of the modules, the amount of information, and participant understanding of the modules will be assessed. Outcomes will be collected on completion of the intervention at 3 weeks, and at a 2-month follow up from trial entry.

**Discussion:**

This trial has the potential to improve CM health literacy in older Australians. There are no educational resources designed to support decision self-efficacy and improve health literacy amongst older people related to CM.

**Trial registration:**

Australian New Zealand Clinical Trials Registry (ANZCTR), ACTRN12616000135415. Registered on 5 February 2016.

**Electronic supplementary material:**

The online version of this article (doi:10.1186/s13063-017-2182-2) contains supplementary material, which is available to authorized users.

## Background

More than three million Australians (14% of the population) are aged over 65 years [1]. Studies indicate seniors take an active role in their health care to improve their health and wellbeing [2], and there is growing evidence that seniors are high users of complementary medicines (CMs). A survey of older Australians found 58% of those aged over 65 years have used at least one of 17 common CM modalities in a previous 12-month period, and 65% of these had visited a CM provider [3]. The Australian Longitudinal Study of Aging, an ongoing prospective study of the older population, demonstrated the prevalence of CM utilisation to have increased over time, from 17% in 2000–2001 to 35% in the 2003–2004 period [4]. CM is generally used to treat a wide range of chronic health complaints that become increasingly common with age [5], particularly musculo-skeletal conditions and pain [6], and anxiety or depression [7]. CM users report significant benefits to both their physical and mental health from CM [8, 9], and that CM is highly valued by encouraging individuals to participate in their health [10].

To be health literate implies having a range of skills and knowledge about health and health care, including the ability to find, understand, interpret, and communicate health information, seek appropriate care, and make critical health decisions [11]. Systematic reviews have concluded that low levels of health literacy are associated with poorer treatment outcomes, including poor compliance with medication, increased admissions to emergency departments, lower ability to interpret labels and health messages, reduced health status, and increased mortality among the elderly [12]. The ability to obtain reliable health information is important to all populations but particularly older people. Reports indicate an increasing number of older people are seeking information on the Internet [13], yet remain unaware of how to identify quality information to guide their use of this information.

There has been significant discussion in the literature about what constitutes health literacy and how to measure it. The health literacy Ophelia Project undertaken at Deakin University, Australia, identified nine concepts of health literacy [14]. These include having sufficient information to manage health, social support for health, skills to appraise health information, ability to engage with healthcare providers, capacity to navigate the healthcare system, ability to find good health information, and sufficient understanding of health information to know what to do with it.

Complementary medicine may offer a way for older people to cope with their ill health, or to engage in maintaining their health; however, this assumes good health literacy skills. There has been little research examining the decision self-efficacy of CM users and their levels of health literacy. This could be of particular concern due to the use of CM without adequate supervision by a qualified health practitioner, and a higher prevalence of polypharmacy arising from the treatment of complex chronic health conditions [15]. Seniors may also be more susceptible to medication sensitivity due to less optimal organ function associated with aging [15–17]. Together, these concerns may increase the risk of potential CM-drug interactions. In the Australian community, the prevalence of serious adverse reactions to CM is relatively low compared to pharmaceutical medications [18]; however, mild reactions are common. A retrospective review of previously collected health data identified that 15% of 5052 participants used CM and 5.8% were identified as having a significant risk of an adverse reaction [19]. These risks were linked to garlic and ginkgo biloba and their interactions with drugs affecting blood coagulation, such as aspirin and warfarin, which, represented 95% of the (potential) significant interactions identified. Other high-risk natural product and drug combinations identified as potentially dangerous include ginseng and warfarin, and St John’s wort used along with digoxin.

This risk of interactions is further complicated by limited disclosure of CM use between consumers and their healthcare providers. Non-disclosure rates to healthcare providers among those using CM have been reported to be as high as 71% [18, 20]. There are multiple reasons for non-disclosure including the individual forgetting to mention CM use, disclosure not being seen as relevant, the doctor not asking about CM use, and the doctor not respecting the value of CM [20]. Disclosure and communication about CM is essential for achieving optimal treatment outcomes.

To explore these issues, we recently completed a two-phase, sequential, mixed-method study incorporating quantitative and qualitative methods to examine CM health literacy in a population of older Australians living in retirement villages [21]. We found older Australians using CM were making decisions regarding managing their own health and self-care [21]. Our primary findings suggest this population demonstrate differing competencies relating to health literacy. We identified three scales where older adults scored low including *navigating the health care system, an ability to find good information*, and *appraisal of health information*. Interpretation of these scales suggests that some participants were unable to understand most health information and were confused when they were presented with conflicting information. We found that these seniors were also unable to access health information when required, they were frequently dependent on others to offer information, were unable to advocate on their own behalf, and struggled to find someone who could help them to use the healthcare system to address their health needs.

There are few programmes that have addressed the specific health literacy needs of older people. A pilot health literacy programme that aimed to build health information literacy skills found that following literacy workshops, participants were empowered to ask questions and reported greater success with finding health information online [22].

Based on our study findings we have developed an educational intervention to increase older adults’ skills and ability to identify good and reliable sources of CM information, resolve conflicting information, and access a diverse range of current CM information sources that can be used to guide their CM decisions with their healthcare providers. The aim of this study is to determine the effectiveness of the CM educational intervention to increase older adults’ health literacy and decision self-efficacy. Improved CM health literacy will enhance appropriate use of health services and ultimately enable older Australians to engage in taking an active role in their health, and reducing the potential for adverse health outcomes.

## Methods/designs

The primary hypothesis is that participants receiving a web/DVD plus booklet intervention compared with a booklet-only group will demonstrate an increase in decision self-efficacy. The secondary hypotheses are that participants receiving a web/DVD plus booklet intervention compared with a booklet-only group will demonstrate an increase in health literacy, communication skill, and change in health-seeking behaviour.

### Study design

**Fig. 1:**
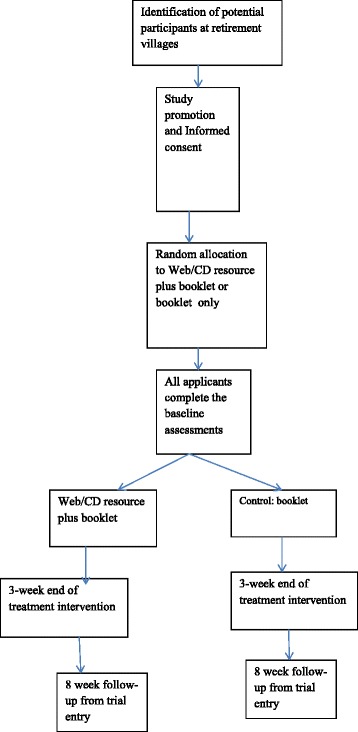
Schematic illustration of the research design

This study (see Fig. 1) is a parallel, randomized, controlled trial of a CM education intervention delivered online (using a purpose built website) or DVD plus booklet versus a control group (booklet only) to examine the effect on decision self-efficacy, health literacy, perception of risk, and health-seeking behavior, on completion of the intervention at 3 weeks, and at 2-month follow up from trial entry. Full details of reporting of the trial protocol items can be found in the “Standard protocol items: recommendation for interventional trials” (SPIRIT) checklist (please see Additional file 1).

### Trial setting

We will recruit participants from a community setting comprising retirement villages and community groups, based in Sydney, Australia. In Australia, a retirement village is made up of housing for people aged over 55 years who are able to live independently. Many of these villages offer some healthcare services, leisure facilities, and social clubs. The village provides smaller, manageable housing, supportive of the changing needs of older people. In this setting, we will contact the managers of the retirement villages to describe the study and seek permission to come and promote the study to residents. We will also recruit from various community groups, including senior citizen clubs, associations and referrals from study participants.

### Study population

The study population will comprise men and women aged 65 years and older, with access to the Internet or a DVD player or computer, who will provide informed consent for participation in the study. Exclusion criteria include being unable to communicate in English or living in a long-term care facility.

### Randomisation and blinding

The randomisation sequence was computer generated by an online randomisation service by Sealed Envelope randomisation service, Ltd 2016 (https://www.sealedenvelope.com/), with the codes concealed in sealed, opaque envelopes. Participants will be randomised in a 1:1 ratio, in blocks of eight to either group 1: web/DVD resource plus booklet, or group 2: control booklet only (see Fig. 1). Participants are not blind to their group allocation; however, the study analyst will be blind during analysis, and the codes will be broken following statistical analysis. The trial co-ordinator was not blind to study group.

### Sample size calculation

The sample size was calculated using GPower, and was based on “sample size effect” drawn from published data [23]. It was estimated that a moderate effect size would be obtained (i.e. Cohen *D* = 0.5) for the primary endpoint only, with improved decision self-efficacy between groups at the end of the 3-week intervention. With the alpha value set at 0.05 and power at 0.8 (i.e. 80% chance that the expected effect size would be significant), a minimum sample size of 64 per group, with rounding to 70 per group was estimated. Allowing for 20% attrition, a total sample size of 168 participants was required for this trial (i.e. 84 per group).

### Interventions

The educational intervention has been informed by our preliminary research [21]. The delivery of the intervention will comprise a multi-media (web/DVD) intervention, and booklet. The delivery format has been informed by findings from the Cochrane systematic review of multimedia educational interventions and offers advantages over the traditional information delivery [24]. A combination of audio with video or graphical presentation can overcome difficulties with low literacy skills [25]. Studies have also shown that learning is improved when material is presented as an audio-visual rather than visual-alone format [26, 27]. A multimedia format also has the advantage that the resources can be used at a pace that suits the user, and at a time and place that is convenient to the individual.

The education intervention comprises five modules, and includes information about CM and other self-care practices, such as exercise. The intellectual content of the intervention has been developed from the Complementary Medicine Education and Outcomes (CAMEO) research programme [28], and modified for use in the Australian context. The CAMEO resource comprises a website and booklet and was developed with links to credible and evidence-based information resources (www.cameoprogram.org/). The CAMEO resource was developed for patients with cancer; however, for this study the education resources have been adapted for use with older individuals.

### Group 1: DVD/web format

The intervention comprises five modules:

1. Module 1 - *Complementary medicine - the evidence.* This module provides scientific evidence related to the health benefits of CM, its indications, and details of various evidence-based CM that are widely practiced globally.
2. Module 2 - *Finding and evaluating complementary medicine evidence*. This module provides an introduction to scientific evidence and how to find research-based studies about CM. It describes databases that are available to find research, how to conduct a search, and how to use the available evidence in making an informed choice about CM.
3. Module 3 - *Decision making – complementary medicine.* In this module, advice is provided regarding how to bring together the information they have obtained from earlier modules, aligning this with their goals and values, and how to have discussions with relevant key people to make an informed decision about the use of CM. The universally employed situation, choices, objectives, people, evaluation, and decisions (SCOPED) framework [29] has been incorporated to assist the participant in their decision making.
4. Module 4 - *Working with complementary medicine practitioners*. This module explains the role of conventional healthcare providers and CM practitioners, and the importance of, and how to disclose CM use with conventional health providers. Guidance is provided regarding the regulatory frame work for CM practitioners in Australia, how to find a professionally accredited CM practitioner and practical tips and questions to ask to guide the selection of a CM practitioner.
5. Module 5 - *Monitoring complementary medicine decisions*. This module explains the need to monitor one’s health following the use of CM products or therapies. It provides guidance on certain criteria that should be utilised in respect to monitor one’s health and the safety of CM and the procedure (including contact details) of adverse events reporting for CM therapy and services in Australia. There is a final section that includes two case studies of individuals exploring self-care and use of CM that draws on the detailed information presented in the modules.

Participants in the intervention group will be invited to watch the five-module intervention in their home by either accessing a website using a password, or viewing a DVD player or computer, over a 3-week time period. Each module takes approximately 30 minutes to complete, and completion of two modules per week over 3 weeks is recommended. Users will be able to pace their learning using the navigation menus on the website or DVD. Participants are able to access the intervention as often as they wish. They will also receive a copy of the booklets.

### Group 2: control group

The active control group comprises two booklets including summarised content from modules one, three, five and the case studies. The content focuses on presenting information on evidence-based CM modalities, guidance to sourcing reliable CM information, how to make decisions about evidence-based CM, why it is important to monitor and evaluate the use of CM, and details about how to discuss CM use with your healthcare provider. A second booklet provides written examples of the two case studies, and applying the information into practice. The booklet text is written in 18-point Arial font and at a 6th-grade reading level. Paced reading is encouraged over the 3-week intervention.

### Development and piloting of the intervention

The investigators undertook development, proofing and editing of the web materials and booklet, with the assistance of web and communication officers at Western Sydney University. Following development of the modules and booklet, a pilot study was undertaken for preliminary testing of the educational and assessment tools. Four participants were presented with the booklet or access to the website to review the booklet or selected web-based modules. Participants were requested to read the allocated resources and to complete a short feedback form on the content of the resources, ease of understanding the material, did they feel overwhelmed by the amount of content, did the images help them to engage with the material, was the font size and layout appropriate, and was the website easy to navigate? The participants were also invited to come together in a focus group context to discuss the resources. The group session was led by GG and the interview guide included questions about ease of use of the educational materials (i.e. font size, colour, background, instructions), and how long it took participants to complete the modules. For those using the website specific questions were asked about where they accessed the website, and what did they use (i.e., tablet, laptop, desktop). Participants were also asked about the ease of understanding the concepts presented.

Feedback from the focus groups indicated participants found the information useful and interesting, they enjoyed the interactive, engaging and colourful format of the resources, the modules did not require as much time as expected to complete, and they enjoyed accessing the resources in their own time. The baseline questionnaire was also pilot tested with the focus group and as a result small wording and skip logic changes were undertaken to the instruments.

### Procedure for the trial

Following completion of the pilot and minor modification to the resources, recruitment for the main trial commenced in July 2016. Recruitment will be undertaken at all interested retirement villages. Study promotion will involve letter box drops, and promotional talks by the investigators and the trial co-ordinator. At these visits, following a promotional study presentation, expressions of interests will be gathered and participant information and consent forms made available. A mutually convenient time will be made to obtain informed consent and to complete baseline questionnaires.

**Fig. 2:**
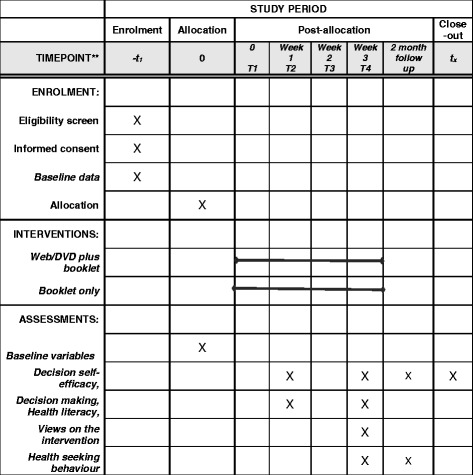
Standard protocol items: recommendation for interventional trials (SPIRIT) schedule of enrolment, interventions, and assessments

Following randomisation, the trial co-ordinator will meet with each participant to ensure they are able to access the website using the password and to navigate the modules, or ensure navigation with the DVD is satisfactory. To minimise attrition, all groups will receive a phone call mid-way through the intervention to ensure they are continuing to access the resources and address any difficulties. Primary and secondary outcome data will be collected at baseline, at the end of the intervention and at two months from trial entry. At trial entry we will collect baseline data on socio-demographic (age, gender, place of birth, education status, employment status, ethnicity, English skills, Medicare and private health insurance) and health characteristics, health behaviour, and lifestyle including CM use, sources of information, Internet skills, health literacy status, and decision-making (Fig. 2: SPIRIT figure).

### Data management

Data capture will be via paper-based questionnaires. Data will be manually entered onto the electronic REDCap data management system by the trial co-ordinator. Any issues will be discussed at the weekly research meeting. All entered data will be de-identified.

### Primary outcome measure

#### Decision self-efficacy

The primary outcome is the change on decision self-efficacy at the end of the intervention assessed between groups. The Decision Self-Efficacy scale [30] will be used to assess differences in decision-making skills. The Decision Self-Efficacy scale will measure self-confidence or belief in one’s ability to make decisions, including an ability to participate in shared decision making. This scale comprises 11 items rated along a 5-point scale from “not at all confident” to “very confident”. The psychometric properties report an alpha coefficient of 0.92, and the scale has been shown to be correlated with decisional conflict scales (DSC) of feeling informed (0.47) and supported (0.45) [32]. Scores on the scale are converted to a 0–100 scale. Scores range from 0 (extremely low self-efficacy) to 100 (extremely high self-efficacy).

### Secondary outcome measures

The preparation for the decision-making scale assesses a participant’s perception of how useful a decision aid or other decision support intervention is in preparing the individual to communicate with their practitioner at a consultation focused on making a health decision [31]. The scale addresses concepts of preparedness for decision making and consists of 10 items rated along a 5-point scale from “not at all” to “a great deal”. This scale has shown significant correlation with the informed (*r* = − 0.21, *p* < 0.01) and support (*r* = − 0.13, *p* = 0.01) DSC subscales [32], and discriminates significantly between participants who did and did not find the decision aid helpful (*p* < 0.0001). Alpha coefficients for internal consistency ranged from 0.92 to 0.96. The scale is strongly unidimensional and item response theory analyses demonstrated that all ten scale items perform well [31]. The scale is only administered after the intervention has been administered, and the items are summed, scored and converted to a 0–100 scale. Higher scores indicate a higher perceived level of preparation for decision making.

The health literacy of participants will be evaluated using a validated and universally employed Health Literacy Questionnaire (HLQ) [14]. The HLQ contains a total 55 questions grouped into nine domains including: (1) feeling understood and supported by healthcare providers; (2) having sufficient information to manage personal health; (3) an ability to actively manage personal health; (4) social support for health; (5) appraisal of health information; (6) ability to actively engage with health care providers; (7) navigating the health care system; (8) ability to find good health information; and (9) understanding health information well enough to know what to do. Each scale includes four to five items, with participants indicating their response along a Likert-type scale with response options ranging from 1 - “very difficult” to 4 - “very easy”, or along a 5-point scale ranging from “strongly agree” to “strongly disagree.” The HLQ has strong psychometric properties, it is grounded in the individual’s lived experience, and is validity driven [14]. Reliability testing was examined using Raykov’s procedures rather than Cronbach’s alpha where > 0.80 was sought. This was achieved for eight of the nine domains; the lowest reliability estimates were achieved for the appraisal of information (0.77). Health literacy scales will be calculated using a scoring algorithm for the HLQ version 1 (dated 2012). The algorithm produces unweighted scale scores for the nine scales of the HLQ, with the final score for each subscale an average score across all items forming the scale. For missing values, this programme uses an algorithm to impute missing values. For scales with four to five items, two missing values can be imputed. For scales with six items, three missing value can be imputed, and if more responses among the scale items were missing, scale score were not computed.

Health-seeking behaviour will be assessed by responses to questions on use of self-care and CM. It will be assessed through a self-reported questionnaire based on questions relating to use of health information sources. Perception of risk will ask questions about the safety of CM. To evaluate the intervention we are seeking participants’ views on the ease of using the resources, the length of the modules, the amount of information and participant understanding of the modules and written information. We will ask participants if they have used the resources outside of the intervention, and if yes, how easy or difficult this was, and did the resources assist with making an informed decision. All data forms will be completed by study participants.

### Statistical analysis

The trial co-ordinator will co-ordinate all data management and cleaning prior to analysis. Data on refusal and dropout will be coded and reported according to Consolidated Standards of Reporting Trials (CONSORT) guidelines [33, 34]. A description of the baseline characteristics of study participants will be compiled using descriptive statistics, such as mean, standard deviation for continuous variables and categorical variables will be summarised by counts and percentages.

The primary analysis will be conducted using all randomised participants. The chi square (*X* ^2^) test and analysis of variance (ANOVA) will be used to identify the differences between intervention groups for the categorical and continuous variables respectively. Secondary analyses will examine changes within group over time using repeated measures ANOVA and the Sidak test to correct for multiple comparisons [35]. Levels of significance will be reported at the *p* < 0.05 level. All CIs will contribute to data interpretation. All analyses were conducted using SPSS statistical software, version 22. The results from this unblended trial will be critically evaluated for bias, and this will be considered during interpretation of the results.

## Discussion

We have identified a need to support older adults’ decision self-efficacy regarding CM. Low levels of health literacy have also been identified among an older population of CM users, who struggle to find good information, and understand what it means [21]. This trial seeks to improve decision making by providing information resources to participants to understand why research studies are important, to seek out reliable information and apply new skills to inform their decision self-efficacy. We aim to examine changes in behaviour in the short term and believe these information resources have the potential to reduce the risk of adverse events arising from mis-information.

We expect that this trial will contribute to our understanding of interventions aimed at supporting decision self-efficacy regarding any self-care behaviour to maintain health and wellbeing, including appropriate use of self-care and CM in older Australians. In addition, by assessing the effect of the intervention on decision self-efficacy, health literacy and health-seeking behaviour, and we will gain new insights into the preferred method of delivery of information resources to older Australians. The outcomes from this study will be important given the high rates of non-disclosure to health professions regarding CM use and previously identified areas of low health literacy.

### Dissemination

We plan to disseminate findings via an academic journal, we will also write to all study participants and retirement villages with a copy of the results. All reports will follow the CONSORT guidelines [36] and the extension to non-pharmacological interventions [34]. We will follow the NHMRC codes of conduct for research authorship. The trial protocol will be made freely available. A reasonable request for de-identified data set will be considered by the investigators.

### Trial status

Recruitment commenced in July 2016. Recruitment was completed in May 2017, and the study was completed in July 2017.

## Additional file

Additional file 1: Spirit checklist. (DOC 120 kb)

## Abbreviations

CM: Complementary medicine
DSC: Decisional conflict scales
HLQ: Health Literacy Questionnaire
